# A survey of the involvement of primary care doctors in HIV prevention and care in a low-prevalence, high-income setting

**DOI:** 10.1186/s12875-021-01376-1

**Published:** 2021-01-28

**Authors:** Greta Tam, Ngai Sze Wong, Shui Shan Lee

**Affiliations:** 1 School of Public Health, The University of Hong Kong, 1/F, Patrick Manson Building (North Wing), 7 Sassoon Road, Pokfulam, Hong Kong; 2 Stanley Ho Centre for Emerging Infectious Diseases, Jockey Club School of Public Health & Primary Care, The Chinese University of Hong Kong, 204-208 Postgraduate Education Centre, Prince of Wales Hospital, Shatin, NT Hong Kong

**Keywords:** HIV, Survey, Primary care

## Abstract

**Background:**

In high-income countries with a low HIV prevalence, primary care doctors are likely the first point of medical contact for people at high risk of HIV. One of the key factors for successful implementation of preventive measures is the cooperation of primary healthcare providers. Hong Kong’s population mostly seek primary care in the private sectors. Our study evaluated the involvement of private primary healthcare providers in HIV prevention and care.

**Methods:**

A cross-sectional postal structured questionnaire was administered to 1102 private primary care doctors in Hong Kong in December 2017. Responses were received via postal mail, fax or online. Non-respondents received a phone-call reminder to complete the survey. Descriptive analyses were performed for all the question items. Chi-square test was used to assess the association between participants’ level of involvement in HIV prevention and care and their demographics and medical practice characteristics.

**Results:**

The response rate was 17.9% (197/1102). Most of the respondents were Chinese (95%) and have obtained their primary medical qualifications in Hong Kong (72%). More than half of the doctors have practiced in the private sector for more than 20 years (54%). Six aspects were used to evaluate practices or involvements in HIV prevention or care: Most of the responding doctors had offered advice (61%) and/or HIV test (76%) to patients with high-risk behaviors. However, fewer doctors had diagnosed HIV (27%), provided care for HIV positive patients (21%), reported HIV cases (19%) or prescribed antiretrovirals (4%). Nine (4.5%) did not answer all six questions on their practices or involvements in HIV prevention or care. The remaining respondents were then categorized into no/low involvement group and high involvement group. Overall,71% had no/low involvement (133/188) compared to 29% who had high involvement (55/188). Factors associated with high involvement included being in the 50–59 age group (OR: 2.48, 95% CI: 1.12–5.5), and belonging to a large practice (OR: 3.16, 95% CI: 1.4–7.12).

**Conclusions:**

Overall, most private primary care doctors in Hong Kong have no or low involvement in HIV prevention and care. However, most were willing and experienced in providing general preventive services, such as HIV testing and advice.

**Supplementary Information:**

The online version contains supplementary material available at 10.1186/s12875-021-01376-1.

## Background

Countries with low HIV prevalence could have significant numbers of undiagnosed persons living with HIV (PLHIV) [1]. This poses a major obstacle to reaching the UNAIDS target of 90-90-90 [2]. High income countries often have good healthcare infrastructure that could potentially provide more comprehensive HIV prevention through proactive testing policies, post-exposure prophylaxis (PEP) and incorporation of new strategies such as providing pre-exposure prophylaxis (PrEP), as recommended by the World Health Organization (WHO) [3]. However, these policies could only be successfully incorporated if healthcare providers have adequate knowledge and willing attitudes.

Hong Kong is an example of a low-prevalence, high-income setting where late diagnosis (AIDS diagnosis within 3 months of HIV diagnosis) in the general population remains a problem, while expanded HIV testing and provision of PrEP have yet to be rolled out [4]. Expanded HIV testing is advocated in outpatient care [5, 6] to decrease the frequent missed opportunities for diagnosis in primary care [7, 8]. The Hong Kong Advisory Council on AIDS currently advocate annual universal HIV testing for all men who have sex with men (MSM) [9]. However, many HIV infected persons in the general population are missed at early stages as they may be reluctant to seek screening in the public service, under the current system [10]. At-risk populations still have inadequate testing rates. Public consultation in Hong Kong revealed requests to increase availability of PrEP and PEP information and services. PrEP has not been introduced into the public service in Hong Kong yet [9]. As there is no national insurance program in Hong Kong, PrEP can only be acquired privately at the cost of USD1000 per month [11]. Meanwhile, PEP is only available in hospitals [9].

Primary care doctors are often the first points of medical contact for the general population, including key populations. Approximately 70% of clinic consultations in Hong Kong are with primary care practitioners in the private sector [12]. Therefore, to identify potential barriers to expanding HIV prevention and care, understanding the current involvement of primary care doctors is necessary. To address this issue, we conducted a survey of private primary care providers in Hong Kong.

## Methods

### Setting and study population

A postal structured questionnaire was administered to private primary care doctors in Hong Kong in December 2017. Contacts of a total of 1102 subjects were obtained from the Primary Care Directory, Medical registrar and Hong Kong College of Family Physicians directory. Responses were received via postal mail, fax or online replies (survey monkey). After 1 month, non-respondents received one reminder phone call offering to re-send the questionnaire to them if necessary.

Ethics approval was obtained from The Chinese University of Hong Kong Survey and Behavioral Research Ethics Committee.

### Questionnaire

The questionnaire was built from items in a literature search using terms related to HIV/AIDS, family practice and involvement. We included qualitative and quantitative studies that referred partially or totally to involvement in HIV prevention and care. We excluded studies that did not involve primary care doctors’ views and those from high prevalence or low-income settings. Three investigators conducted independent searches, with the final retained studies decided by the first author. We retained five studies from the USA [13–17] and one from UK [18].

Content validation was undertaken with three doctors. Two doctors had experience in primary care (one had a specialist qualification), while the remaining doctor was an HIV specialist. The survey was reviewed online by each doctor for relevance and clarity and revisions were made accordingly.

Participants’ demographics and their medical practice characteristic were reported using single-response answers. Attitudes towards HIV prevention and care were categorized using a 3-item Likert scale: (items included disagree, neutral and agree). HIV prevention and care practices were reported using multiple answers and frequencies.

The questionnaire is included as a supplementary file.

### Statistical analysis

Descriptive analyses were performed on all question items. These included frequencies and simple proportions. Following the descriptive analyses, participants’ involvement was categorized based on their responses to six items of HIV prevention and care. Participants were categorized into 4 levels of involvement: None, low (answering “yes” to one or two items), medium (answering “yes” to three or four items) and high (answering “yes” to five or six items). This was then collapsed to a binary outcome variable for ease of interpretation: Participants were categorized into no or low involvement group and high involvement group. Participants who answered “yes” to three or more items were categorized as high involvement, while those who answered “yes” to two or less items were categorized as no or low involvement.

Bivariable logistic regression was conducted to assess the association between participants’ level of involvement in HIV prevention and care and their demographics and medical practice characteristic. Additional analyses were conducted to compare the demographics between respondents that answered immediately and respondents who answered after the reminder and to compare between respondents with no HIV patients and respondents with HIV patients. Statistical significance was classified by *p*-value < 0.05. Data was analyzed using STATA SE 2012.

## Results

Socio-demographic and medical practice characteristics (Table 1).

**Table 1:** Socio-demographic and medical practice characteristics of survey respondents, 2017 (*n* = 195)

	n	%	Total number
Gender			
female	43	22%	
male	152	78%	195
Age group			
30–39	11	6%	
40–49	56	29%	
50–59	56	29%	
> 60	71	37%	194
Ethnicity			
Chinese	186	95%	
Asian	4	2%	
White	4	2%	
Others	1	1%	195
Place of primary medical qualification			
Hong Kong	140	72%	
Others	55	28%	195
Specialist register			
FHKCFP	23	12%	
Others	54	28%	
No	117	60%	194
Number of years in private practice			
> 20 years	106	54%	
11–20 years	53	27%	
6–10 years	22	11%	
0–5 years	14	7%	195
Number of clinicians in your practice			
<=5	163	84%	
6–10	12	6%	
11–20	7	4%	
>=20	12	6%	194

The response rate was 17.9% (197/1102). Two respondents (1%) did not complete any of the demographic questions.

Respondents were predominantly Chinese in ethnicity (95%) and their most common place of primary medical qualification was Hong Kong (72%).

Most had 5 or less clinicians in their practice (84%).

### Attitudes towards HIV prevention and care (Table 2)

Most respondents were comfortable taking a sexual history (68%) and the vast majority were willing to provide HIV testing if patient requested (90%). However, less than half were comfortable having an informed discussion with patients regarding PrEP (47%) or were willing to prescribe PrEP to patients in need (34%).

**Table 2:** Attitudes towards HIV prevention and care

	n	%	Total number
Feel comfortable taking a sexual history			
disagree	24	12%	
neutral	38	19%	
agree	134	68%	196
Willing to prescribe HIV testing if patient requests			
disagree	6	3%	
neutral	13	7%	
agree	176	90%	195
Feel comfortable having an informed discussion with patients regarding PrEP			
disagree	41	21%	
neutral	61	31%	
agree	92	47%	194
Willing to prescribe PrEP to patients in need			
disagree	90	46%	
neutral	39	20%	
agree	65	34%	194
PrEP is effective in preventing HIV infection			
disagree	17	9%	
neutral	73	39%	
agree	98	52%	188

### HIV prevention and care practices (Table 3)

Most respondents had experience offering HIV testing (76%) and advice to patients at risk of HIV (61%). However, most had never made any HIV diagnosis before (73%), provided care to HIV patients (79%), reported an HIV case (81%) or prescribed antiretroviral (96%). Most had been involved in HIV prevention and care practices in some way (85%). Regarding their experience with PrEP, most had not prescribed PrEP before (93%).

**Table 3:** HIV prevention and care practices

	n	%	Total number
**Offered advice to patients at risk of HIV**			
	121	61%	197
**Offered HIV testing**			
yes	148	76%	196
if yes, please indicate which of the following:			
*Patients with STI*	112	92%	122
*Antenatal testing*	29	24%	122
*MSM*	44	36%	122
*Patients with TB*	114	93%	122
**Diagnosed HIV**			
	53	27%	197
**Provided care for HIV positive patients**			
yes	40	21%	190
if yes, how many:			
1–10	37	93%	
11–20	0	0%	
21–50	3	7%	40
**Reported HIV case**			
	38	19%	197
**Prescribed antiretroviral**			
yes	8	4%	197
**I have done none of the above**			
disagree	165	85%	
agree	28	15%	193
**Asked about PrEP by patients**			
yes	21	11%	194
if yes, how many times:			
1–5	14	67%	
6–10	5	24%	
> 10	2	10%	21
**Ever prescribed PrEP**			
yes	14	7%	194
if yes, how many times:			
1–5	11	79%	
6–10	2	14%	
> 10	1	7%	14

### Association of clinician characteristics with the level of involvement in HIV prevention and care (Table 4)

Most respondents had no/low involvement in HIV prevention and care (71%). Those aged 50–59 years old tended to have higher involvement (OR: 2.48, 95% CI: 1.12–5.5). In the analyses, place of primary medical qualification, possessing a specialist qualification and number of years in private practice were not associated with involvement. Respondents with more than 5 clinicians in their practice tended to have higher degree of involvement (OR: 3.16, 95% CI: 1.4–7.12).

**Table 4:** Association of clinician characteristics with the level of involvement in HIV prevention and care

	No/low involvement (*n*=133)		High involvement (*n*=55)			
	n	%	n	%	OR	95% C.I.
Gender						
female	31	23%	11	20%	ref	
male	102	77%	44	80%	1.22	0.56–2.63
Age group						
> 60	55	42%	15	27%	ref	
30–39	6	5%	5	9%	3.06	0.82–11.4
40–49	40	30%	14	25%	1.28	0.56–2.96
50–59	31	23%	21	38%	2.48	1.12–5.5*
Ethnicity						
non-Chinese	2	2%	7	13%	ref	
Chinese	131	98%	48	87%	0.10	0.02–0.52*
Place of primary medical qualification						
Other	37	28%	16	29%	ref	
Hong Kong	96	72%	39	71%	0.94	0.47–1.88
Specialist register						
No	86	65%	28	52%	ref	
FHKCFP	13	10%	10	19%	2.36	0.93–5.98
Other	34	26%	16	30%	1.45	0.7–3.0
Number of years in private practice						
> 20 years	71	53%	33	60%	ref	
11–20 years	38	29%	13	24%	0.74	0.35–1.56
6–10 years	15	11%	5	9%	0.72	0.24–2.14
0–5 years	9	7%	4	7%	0.96	0.27–3.33
Number of clinicians in your practice						
<=5	118	89%	40	73%	ref	
> 5	14	11%	15	27%	3.16	1.4–7.12*

Odds ratio (OR): the odds of high involvement in HIV prevention and care compared with no/low involvement in non-reference and reference group

* *p*-value < 0.05

When practice size was controlled for, the association between age group and involvement remained unchanged. When age group was controlled for, the association between practice size and involvement also remained unchanged.

### A comparison of demographics between respondents that answered immediately and respondents who answered after the reminder (Table 5)

In general, demographics of respondents that answered immediately and respondents who answered after the reminder did not differ significantly, with the exception of age. A higher proportion of respondents that answered immediately belonged to the 40–49 years old age group compared to respondents who answered after the reminder (OR 4.09, 95% C.I. 1.29–12.97).

**Table 5:** A comparison of demographics between respondents that answered immediately and respondents who answered after the reminder

	Responded after reminder		Immediate reply			
	n	%	n	%	OR	95% C.I.
Gender						
female	5	14%	38	24%	ref	
male	31	86%	121	76%	0.51	0.19–1.41
Age group						
> 60	17	47%	54	34%	ref	
50–59	11	31%	45	28%	1.29	0.55–3.03
40–49	4	11%	52	33%	4.09	1.29–12.97*
30–39	4	11%	7	4%	0.55	0.14–2.11
Ethnicity						
non-Chinese	0	0%	9	6%	/	
Chinese	36	100%	150	94%		
Place of primary medical qualification						
Other	9	25%	46	29%	ref	
Hong Kong	27	75%	113	71%	0.82	0.36–1.88
Specialist register						
No	21	58%	96	61%	ref	
^a^FHKCFP	5	14%	18	11%	0.79	0.26–2.36
Other	10	28%	44	28%	0.96	0.42–2.21
Number of years in private practice						
> 20 years	22	61%	84	53%	ref	
11–20 years	6	17%	47	30%	2.05	0.78–5.42
6–10 years	6	17%	16	10%	0.70	0.24–1.99
0–5 years	2	6%	12	8%	1.57	0.33–7.54
Number of clinicians in your practice						
<=5	31	89%	132	83%	ref	
> 5	4	11%	27	17%	1.59	0.52–4.86

^a^ Fellow of the Hong Kong College of Family Physicians

*Statistically significant

### A comparison between respondents who saw 0 HIV patients and respondents who saw at least 1 patient (Table 6)

Respondents with no HIV patients tended to be older, with a smaller proportion possessing a specialist qualification (OR 3.54, 95% C.I. 1.33–9.39) or belonging to a large practice (OR 2.54, 95% C.I 1.10–5.88) when compared to respondents with HIV patients.

**Table 6:** A comparison between respondents with no HIV patients and respondents with HIV patients

	Respondents with no HIV patients		Respondents with HIV patients			
	n	%	n	%	OR	95% C.I.
Gender						
female	33	21%	10	25%	ref	
male	122	79%	30	75%	0.81	0.36–1.83
Age group						
> 60	66	43%	5	13%	ref	
50–59	39	25%	17	43%	5.75	1.97–16.82*
40–49	42	27%	14	35%	4.40	1.48–13.11*
30–39	7	5%	4	10%	7.54	1.64–34.77*
Ethnicity						
non-Chinese	4	3%	5	13%	ref	
Chinese	151	97%	35	88%	0.19	0.05–0.73*
Place of primary medical qualification						
Other	41	26%	14	35%	ref	
Hong Kong	114	74%	26	65%	0.67	0.32–1.40
Specialist register						
No	99	64%	18	45%	ref	
^a^FHKCFP	14	9%	9	23%	3.54	1.33–9.39*
Other	41	27%	13	33%	1.74	0.78–3.88
Number of years in private practice						
> 20 years	87	56%	19	48%	ref	
11–20 years	43	28%	10	25%	1.06	0.46–2.49
6–10 years	15	10%	7	18%	2.14	0.77–5.96
0–5 years	10	6%	4	10%	1.83	0.52–6.47
Number of clinicians in your practice						
<=5	134	87%	29	73%	ref	
> 5	20	13%	11	28%	2.54	1.10–5.88*

*Statistically significant

## Discussion

Our results suggest that private primary care doctors in Hong Kong tend to have no or low involvement in HIV prevention and care. This is not surprising as only 14.4% of HIV cases are diagnosed in private clinics in Hong Kong [4]. However, it is difficult to conclude whether the low number of cases diagnosed in private clinics is due to the low involvement of private primary care doctors in HIV prevention and care or vice versa. Patients might have preference for Non-governmental organization (NGO) or public counselling service for HIV testing.

This situation is not unique to Hong Kong. In UK, only 8% of HIV diagnoses made outside STI clinics were those in primary care [19] and only 3.8% of all HIV tests in the country were conducted in primary care [20].

Respondents aged 50–59 had the highest involvement, possibly due to the timing of the HIV epidemic. The first HIV case in Hong Kong occurred in 1984 [21], when this cohort was between 14 and 23 years old. They may have been taught about HIV in medical school and are old enough to have gathered decades of experience in clinical practice. Respondents in large practices had the highest involvement possible due to access to more resources and a higher likelihood of having a specialist in their practice. A comparison between respondents with no HIV patients and respondents with HIV patients revealed similar results, with the addition that respondents with HIV patients tended to have a specialist qualification, when compared to respondents with no HIV patients.

In general, private primary care doctors have experience in HIV prevention and are willing to provide general preventive services, such as HIV testing and advice. However, they have less experience in follow-up HIV care and PrEP prescription and are less willing to provide services in these areas.

Willingness to prescribe HIV testing on request (90% agree, 7% neutral) was higher compared to a survey of primary care doctors in USA (81% agree) [14]. The vast majority of respondents in our study had offered HIV testing to patients seeking treatment for STD (92%) and patients initiating treatment for TB (93%). Although patients seeking treatment for STD were also most commonly offered HIV testing in USA (74%), patients initiating treatment for TB were much less commonly offered HIV testing (36%), while pregnant women and MSM were more commonly offered testing (55 and 52% respectively) [14]. The differences in population-specific testing might be explained by the differences in TB burden and health-care seeking behaviors in the two places. Hong Kong has an intermediate TB burden while USA has a low TB burden [22], therefore the likelihood of encountering a patient initiating treatment for TB would be higher in Hong Kong. Meanwhile, antenatal care is free in the public healthcare service with opt-out HIV screening done in Hong Kong [23], therefore pregnant women might be less likely to present to private primary care doctors for care.

PrEP is not available in the public healthcare system in Hong Kong [11], yet surprisingly some of the respondents had prescribed it. In USA 26% of primary care doctors were reported to have experience prescribing antiretrovirals, compared to only 4% in Hong Kong [14]. Overall, less than 10% have experience prescribing PrEP in Hong Kong (7%) and USA [15]. HIV care is free of charge in the public healthcare system in Hong Kong [24], therefore patients may preferentially seek care there. In contrast, the lack of PrEP availability in the public healthcare system may lead to both a demand and supply in the private primary care sector. The low rates of willingness to prescribe PrEP (34% agree, 20% neutral) in Hong Kong compared to USA (91% agree) [14] indicate that there may only be a small number of practitioners who are willing and able to fulfill the demand for PrEP prescription.

Low HIV prevalence countries should prioritize targeting the undiagnosed population to decrease HIV transmission. A cost-effectiveness study in a low prevalence country concluded that targeted HIV testing of high-risk populations was the most cost-effective strategy and suggested linking HIV testing to other health checks to reduce potential stigma or discrimination [1]. This strategy could be aapplied to Hong Kong to expand HIV testing. While opt-out testing guidelines exist in Europe and USA [25], these have not been established in Hong Kong. However, in addition to being less cost-effective than targeted HIV testing, adherence to opt-out testing was low in Europe and USA, with provider test offer of only 40% [25]. Meanwhile, patient acceptance rate of opt-out testing was only 44% in a study in Hong Kong [26]. Expanding HIV testing by offering opt-out testing of high-risk populations could be a feasible strategy in Hong Kong. As most of our respondents had experience testing patients seeking treatment for STD and patients initiating treatment for TB, encouraging them to expand HIV testing through opt-out testing of these populations may be possible. Opt-out HIV testing of MSM would also potentially reduce the undiagnosed population substantially. However, this would require the patient to disclose their status before the provider could offer HIV testing. Health promotion at private primary care clinics might be needed to increase willingness of MSM to come forward for testing and for providers to be prepared to offer testing to this population.

The implications of our findings can be applied to Hong Kong and to a wider extent, other low HIV prevalence countries. Low HIV prevalence countries might consider switching their testing strategy from opt-out testing to targeted testing. However, the groups to be targeted may differ between countries and settings. The lack of experience in prescribing PrEP amongst private primary care doctors is not unique to Hong Kong. However, as rates of willingness differ between countries, those with high rates of willingness may consider training private primary care doctors to prescribe PrEP.

### Strengths and limitations

This study had several strengths, including: Cross-checking multiple databases to reach all private primary care doctors in Hong Kong and to maximize sample size; non-respondents receiving a follow-up reminder phone call to increase response rate. In addition, multiple channels for responses were made available, including postal mail, fax or online. Nevertheless, despite our best efforts, response rate was still low. This is in line with other studies which surveyed medical providers which achieved response rates of 11–50% [17]. The low rate of survey completion might be a source of bias as those with higher involvement in HIV prevention and care might be more likely to complete the survey. Therefore, the results of this survey may not be fully representative of private primary care doctors’ involvement in HIV prevention and care in Hong Kong. Although we did not have data on non-responders, our comparison of demographics between respondents that answered immediately and respondents who answered after the reminder showed that there was no significant difference, with the exception of one age group. Time and financial constraints prevented us from conducting qualitative interviews to better inform the development of our survey. Further qualitative research is needed to give a more in-depth view and to elaborate the reasons behind our quantitative findings.

## Conclusion

Our survey results indicate that overall, most private primary care doctors in Hong Kong have no or low involvement in HIV prevention and care. However, most were willing and experienced in providing general preventive services, such as HIV testing and advice. Although primary care doctors were less willing nor experienced in providing HIV follow-up care and PrEP, yet a low but substantial proportion had provided PrEP prescription, indicating the demand for PrEP might be currently met through private primary care doctors. Expanding HIV testing through opt-out testing of high-risk populations by private primary care doctors may be a feasible strategy to decrease HIV transmission in low prevalence settings such as Hong Kong.

## Supplementary Information

**Additional file 1.** Study questionnaire. Survey of private primary care practitioners regarding knowledge, attitudes and practices towards HIV Pre-Exposure Prophylaxis (PrEP).

## Data Availability

The datasets analyzed during the current study are available from the corresponding author on reasonable request.

## Abbreviations

PLHIV: Persons living with HIV
PrEP: Pre-exposure prophylaxis
PEP: Post-exposure prophylaxis
WHO: World Health Organization
STD: Sexually transmitted diseases
TB: Tuberculosis
FHKCFP: Fellow of the Hong Kong College of Family Physicians
NGO: Non-governmental organization
