# Supplementary material for: A survey of the involvement of primary care doctors in HIV prevention and care in a low-prevalence, high-income setting
Source: BMC Fam Pract. 2021 Jan 28;22:27. doi: 10.1186/s12875-021-01376-1 (PMC7842046; doi:10.1186/s12875-021-01376-1)
Supplement: Supplementary file 1 — Additional file 1. Study questionnaire. Survey of private primary care practitioners regarding knowledge, attitudes and practices towards HIV Pre-Exposure Prophylaxis (PrEP). [file 12875_2021_1376_MOESM1_ESM.pdf]

Survey of private primary care practitioners regarding knowledge, attitudes and practices towards HIV Pre-Exposure Prophylaxis (PrEP)

This survey aims to collect your views on the role of primary care doctors in HIV prevention. Completion of the questions requires no more than 10 to 15 minutes. Results of this survey would be used for global analysis only without referral to individual respondent.

Please kindly complete and return the questionnaire using one of the following methods:

1) Fax: **2606 3791/2606 3500**, Att.: **Prof. Greta Tam**

2) Mail: A return address label is attached with the following address:

Prof Greta Tam

Rm 404, JC School of Public Health and Primary Care,

Prince of Wales Hospital,

Hong Kong

3) Online: Visit the survey website: **https://www.surveymonkey.com/r/HKDrPrEP**

Or scan the QR code below. Please enter the survey number written at the top of the next page.

If you have any question regarding the study, please contact Prof. Greta Tam of the Chinese University of Hong Kong (Phone: **2252-8799**, Email: **gretatam@cuhk.edu.hk**)

Thank you for your cooperation.

Prof. Greta Tam

Research Assistant Professor, Jockey Club School of Public Health and Primary Care,

The Chinese University of Hong Kong

Survey Number:______________

Basic information

1. Gender:

○ Male ○ Female

2. Ethnicity

○ Chinese ○ Asian ○ White ○ Other _____________

3. Age:

○ <29 ○ 30-39 ○ 40-49 ○ 50-59 ○ >60

4. Place of primary medical qualification:

○ Hong Kong ○ Other ____________

5. Are you on a specialist register?

○ No ○ FHKCFP ○ Other ___________________

6. Type of practice:

○ Solo ○ Multispecialty ○ Single specialty

7. Number of years in private practice:

○ >20 ○ 11-20 ○ 6-10 ○ 0-5

8. Number of clinicians in your practice:

○ ≤5 ○ 6-10 ○ 11-20 ○ ≥20

9. Location of clinic:

○ Central & Western ○ Wan Chai ○ Eastern ○ Southern
○ Yau Tsim Mong ○ Sham Shui Po ○ Kowloon City ○ Wong Tai Sin
○ Kwun Tong ○ Kwai Tsing ○ Tsuen Wan ○ Tuen Mun
○ Yuen Long ○ North ○ Tai Po ○ Sha Tin
○ Sai Kung ○ Islands

Your knowledge of HIV prevention

Pre-exposure prophylaxis (or PrEP) is when people at very high risk for HIV take HIV medicines daily to lower their chances of getting infected.

10. How familiar are you with PrEP in your practice
    - ○ This is the first time I hear about it
    - ○ I have heard about it but not familiar with the actual procedure
    - ○ I am familiar with the principles but have never prescribed
    - ○ I am familiar with the principles and have prescribed PrEP before

Your practices regarding HIV prevention

11. Which of the following have you ever done? (Multiple answers allowed)
    - ☐ Offered advice to patients at risk of HIV
    - ☐ Offered HIV test (if yes, please indicate which of the following:)
        - ○ Patients seeking treatment for STD (sexually transmitted diseases)
        - ○ Pregnant women
        - ○ MSM (men who have sex with men)
        - ○ Patients initiating treatment for TB (tuberculosis)
        - ○ Others ________________________________
    - ☐ Diagnosed HIV
    - ☐ Provided care for HIV-positive patients (if yes, how many?)
        - ○1–10 ○11–20 ○21–50 ○>50
    - ☐ Reported HIV case
    - ☐ Prescribed antiretroviral
    - ☐ None of the above

11. Have you ever prescribed or referred a patient for PrEP? (if yes, proceed to question 12; if no, proceed to question 13)

    ○ Yes ○ No

12. How many times have you ever prescribed PrEP?

    ○ 1-5 ○ 6-10 ○ >10

13. Have you ever been asked about PrEP by patients? (if yes, proceed to question 14; if no, proceed to question 15)

    ○ Yes ○ No

14. How many times have you ever been asked about PrEP?

    ○ 1-5 ○ 6-10 ○ >10

Do you agree with the following statements?

15. I feel comfortable taking a sexual history (*strongly disagree*= 1 <-> *strongly agree* = 5)

    ○1 ○2 ○3 ○4 ○5

16. If a patient requested HIV testing, I would provide it to them
    (*strongly disagree*= 1 <-> *strongly agree* = 5)

    ○1 ○2 ○3 ○4 ○5

17. I feel comfortable having an informed discussion with patients regarding PrEP
    (*strongly disagree*= 1 <-> *strongly agree* = 5)

    ○1 ○2 ○3 ○4 ○5

18. I feel comfortable prescribing PrEP to patients in need
    (*strongly disagree*= 1 <-> *strongly agree* = 5)

    ○1 ○2 ○3 ○4 ○5

19. PrEP is effective in preventing HIV infection
    (*strongly disagree*= 1 <-> *strongly agree* = 5)

    ○1 ○2 ○3 ○4 ○5

20. Risk compensation (increased in the practice of unprotected sex) is not common after PrEP
    (*strongly disagree*= 1 <-> *strongly agree* = 5)

    ○1 ○2 ○3 ○4 ○5

21. The Government should offer PrEP to clients free
    (*strongly disagree*= 1 <-> *strongly agree* = 5)

    ○1 ○2 ○3 ○4 ○5
